# Grapevine (*Vitis vinifera*) responses to salt stress and alkali stress: transcriptional and metabolic profiling

**DOI:** 10.1186/s12870-022-03907-z

**Published:** 2022-11-14

**Authors:** Xu Lu, Lei Ma, CongCong Zhang, HaoKai Yan, JinYu Bao, MeiShuang Gong, WenHui Wang, Sheng Li, ShaoYing Ma, BaiHong Chen

**Affiliations:** 1 College of Horticulture, Gansu Agricultural University, Lanzhou, 730070 China; 2 Agronomy College, Gansu Agricultural University, Lanzhou, 730070 China; 3 College of Life Science and Technology, Gansu Agricultural University, Lanzhou, 730070 China; 4 Basic Experimental Teaching Center, Gansu Agricultural University, Lanzhou, 730070 China; 5 College of HorticultureCollege of Life Science and Technology, State Key Laboratory of Aridland Crop Science, Gansu Agricultural University, Lanzhou, 730070 China

**Keywords:** Transcriptome, Metabolome, Alkali stress, Salt stress, Grapevine plant

## Abstract

**Background:**

Soil salinization and alkalization are widespread environmental problems that limit grapevine (*Vitis vinifera* L.) growth and yield. However, little is known about the response of grapevine to alkali stress. This study investigated the differences in physiological characteristics, chloroplast structure, transcriptome, and metabolome in grapevine plants under salt stress and alkali stress.

**Results:**

We found that grapevine plants under salt stress and alkali stress showed leaf chlorosis, a decline in photosynthetic capacity, a decrease in chlorophyll content and Rubisco activity, an imbalance of Na^+^ and K^+^, and damaged chloroplast ultrastructure. Fv/Fm decreased under salt stress and alkali stress. NPQ increased under salt stress whereas decreased under alkali stress. Gene Ontology (GO) and Kyoto Encyclopedia of Genes and Genomes (KEGG) enrichment showed the differentially expressed genes (DEGs) induced by salt stress and alkali stress were involved in different biological processes and have varied molecular functions. The expression of stress genes involved in the ABA and MAPK signaling pathways was markedly altered by salt stress and alkali stress. The genes encoding ion transporter (AKT1, HKT1, NHX1, NHX2, TPC1A, TPC1B) were up-regulated under salt stress and alkali stress*.* Down-regulation in the expression of numerous genes in the ‘Porphyrin and chlorophyll metabolism’, ‘Photosynthesis-antenna proteins’, and ‘Photosynthesis’ pathways were observed under alkali stress. Many genes in the ‘Carbon fixation in photosynthetic organisms’ pathway in salt stress and alkali stress were down-regulated. Metabolome showed that 431 and 378 differentially accumulated metabolites (DAMs) were identified in salt stress and alkali stress, respectively. L-Glutamic acid and 5-Aminolevulinate involved in chlorophyll synthesis decreased under salt stress and alkali stress. The abundance of 19 DAMs under salt stress related to photosynthesis decreased. The abundance of 16 organic acids in salt stress and 22 in alkali stress increased respectively.

**Conclusions:**

Our findings suggested that alkali stress had more adverse effects on grapevine leaves, chloroplast structure, ion balance, and photosynthesis than salt stress. Transcriptional and metabolic profiling showed that there were significant differences in the effects of salt stress and alkali stress on the expression of key genes and the abundance of pivotal metabolites in grapevine plants.

**Supplementary Information:**

The online version contains supplementary material available at 10.1186/s12870-022-03907-z.

## Background

Soil salinization and alkalization are major factors that seriously affect crop growth and yield globally [1]. It was reported that an estimated 1.13 billion hectares of land worldwide are affected by saline-alkali stress, accounting for more than 20% of the total cultivated area [2]. Although soil salinization and alkalization often occur concurrently in nature, alkaline stress and salt stress in saline-alkalization soil are two distinct types of stress faced by plants [3]. Salt stress caused by neutral salts, such as NaCl and Na_2_SO_4_ [4], leads to osmotic stress and ionic imbalance in plants, thereby negatively affecting the metabolism process [5]. Alkaline stress caused by NaHCO_3_ and Na_2_CO_3_ has higher pH than salt stress, which results in plants confronted with not only the same osmotic stress and ionic toxicity caused by salt stress, but also high pH stress. Therefore, alkaline stress is a more diverse form of stress and is more harmful to plants compared with salt stress.

Currently, the research on the effect of saline-alkali stress on plant growth mainly focuses on salt stress but alkaline stress attracts less attention. Many plants respond to salt or alkali stress by regulating signal transduction, ion homeostasis, a series of metabolic pathways (such as photosynthesis and organic acid metabolism), and resistance gene expression [4, 6]. Plant hormone signaling [7] and MAPK signaling pathway [8] are responsible for sensing and responding to environmental stresses, thereby triggering major changes in gene expression and adaptive physiological responses. Furthermore, many genes involved in the two signaling pathways played a crucial role in salt stress defense, such as *PtSnRK2.5*, *PtSnRK2.7*, *PP2C* [9, 10], *MsCML46* [11], and *MPK3* gene [12].

Intracellular ion balance is disturbed by saline-alkali stress, leading to the disorder of biological processes such as photosynthesis. Thus, maintaining the Na^+^ and K^+^ homeostasis is pivotal for plant survival in saline-alkali environments. The key genes encoding Na^+^ and K^+^ transporters have been identified in plants. The *NHXs* and *HKT1* genes are involved in plant response to salinity and their abundance can improve crop salt resistance [13–15]. Particularly, *NHX1*-*NHX8* plays a pivotal role in maintaining Na^+^ homeostasis through intracellular Na^+^ excretion and vacuolar Na^+^ compartmentalization [13, 16]. However, there were fewer reports of genes encoding Na^+^ and K^+^ transporters in plants under alkali stress, and previous studies mainly focused on salt stress. In addition to ion transporters, secretion of organic acids (OAs) in saline-alkali environments also helps plants maintain ion balance and intracellular pH stability in response to environmental changes [17–19].

The reduction of plant photosynthetic capacity caused by salinity stress is directly related to the reduction of yield. Therefore, investigating detailed information about plant responses to stress and the adaptation strategies employed by them to save their photosynthetic apparatus could be helpful to develop new crops with more robust photosynthetic machinery for higher yields even in stressed environments [20]. Stomatal conductance, chlorophyll biosynthesis, activity of Rubisco and other key enzymes, chloroplast ultrastructure, electron transport, non photochemical dissipation of heat energy and changes gene expression, are crucial to the operation of photosynthesis [21]. However, the effects of alkali stress on these factors are rarely reported.

Grapevine (*Vitis vinifera* L.) has high economic value and is considered one of the world’s most important fruit crops [22]. Grapevine not only is used to produce wine but is also consumed fresh and processed into raisins and juice. Most grapevine-growing regions are in arid or semi-arid areas [23]. In these areas, soil salinization and alkalization are a concern due to low rainfall and high evaporation [24]. Although grapevines could grow in this saline-alkali environment, in the soil affected by saline-alkali, grapevine roots often accumulate a large amount of salt, and root vitality also decreased caused by high pH, which seriously affects the normal growth and development of grapevine plants, resulting in yield loss and resulting in yield loss and quality change [25, 26]. Therefore, soil salinization-alkalization is the key factor limiting the growth and yield of grapevine.

Several studies have been reported on the physiological characteristics and transcript changes of grapevine plants under salt stress [27–30]. However, information on the transcriptional and metabolic profiling of grapevine plants in response to alkaline stress is unclear. Knowledge of the differences in genes and metabolites that maintain ion homeostasis and photosynthetic function in grapevine plants under salt stress and alkali stress is particularly limited. Here, we investigate the physiological characteristics, chloroplast ultrastructure, comparative transcriptomic and metabolomic on grapevine plant cuttings exposed alone to salinity stress and alkali stress, which could provide new insight into the differences in the response of grapevine plants to salt stress and alkali stress.

## Results

### Salt stress and alkali stress affected phenotype and physiological characteristics in grapevine plant

**Fig. 1:**
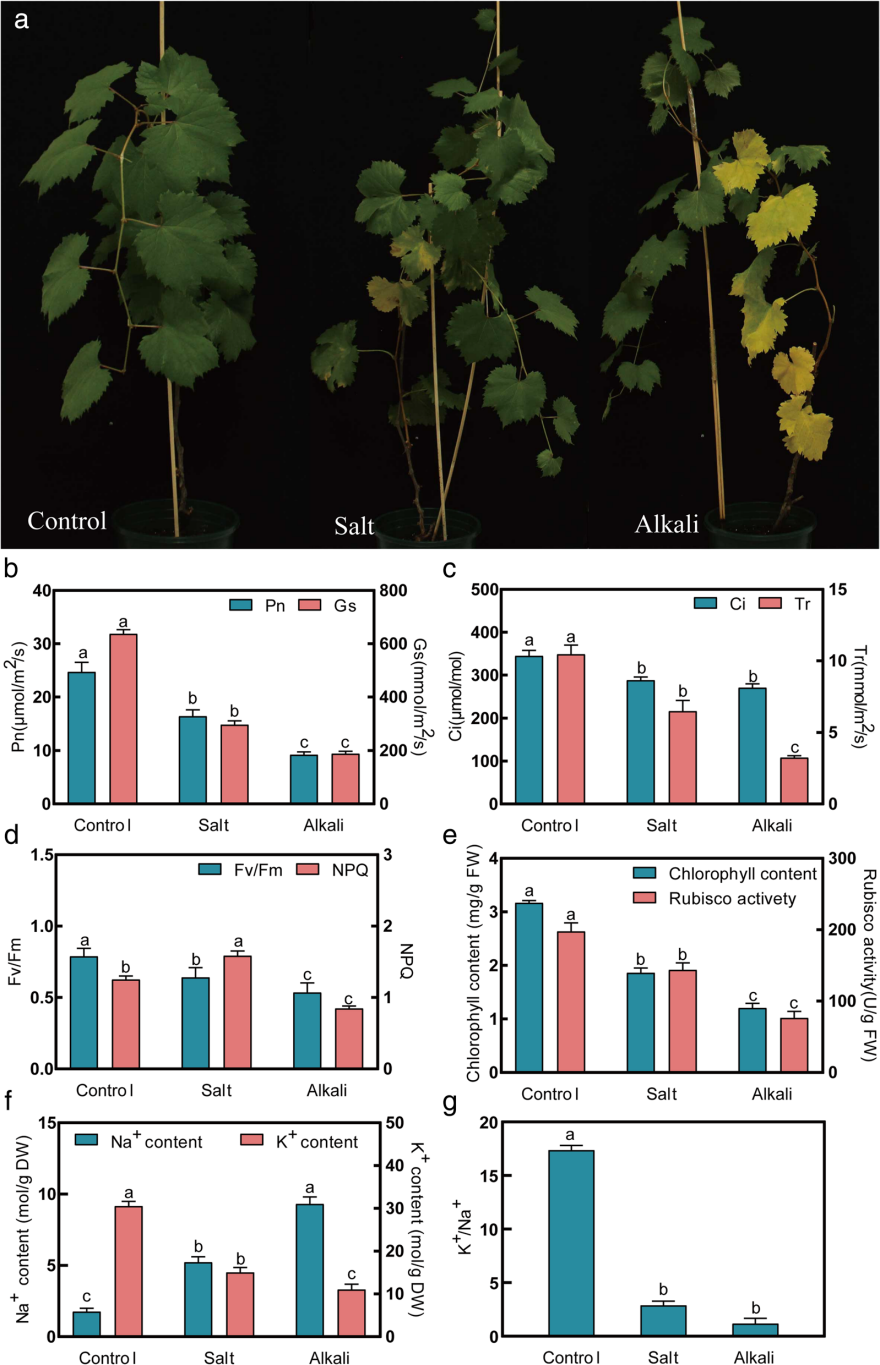
The phenotype and the value of physiological parameters of grapevine plants under Control, salt stress, and alkali stress conditions after 20 days. **a** The phenotype of grapevine leaves. **b** Pn and Gs. **c** Tr and Ci. **d** Fv/Fm and NPQ. **e** Chlorophyll content and Rubisco activity. **f** Na^+^ and K^+^ content. **g** Ratio of Na^+^/K^+^. Lowercase letters represent significant differences among sampling treatments (*p* < 0.05)

Stress symptoms and leaf damage in grapevine plants exposed to stress for 20 days were observed. Following salt stress and alkali stress treatment, older leaves showed chlorosis. Alkali stress induced more etiolated leaves than that salt stress (Fig. 1a). We measured physiological parameters to investigate the effects of salt stress and alkali stress on grapevine plants. Significantly, gas exchanged parameters (Pn, Gs, Tr, and Ci) markedly decreased in plants subjected to salt stress and alkali stress compared to control plants (Fig. 1b and c). The Fv/Fm was significantly lower under salt stress and alkali stress (Fig. 1d). However, NPQ was increased under salt stress relative to control but was decreased under alkali stress (Fig. 1d). The content of chlorophyll and Rubisco activity was decreased under two stress compared with control (Fig. 1e). The Na^+^ content in leaf increased, whereas K^+^ content decreased in response to salt stress and alkali stress compared to control (Fig. 1f), thus raising Na^+^/K^+^ ratio (Fig. 1g). Notably, alkali stress resulted in more bad effects on these physiological properties than salt stress.

### Salt stress and alkali stress destroyed chloroplast ultrastructure in grapevine plant

**Fig. 2:**
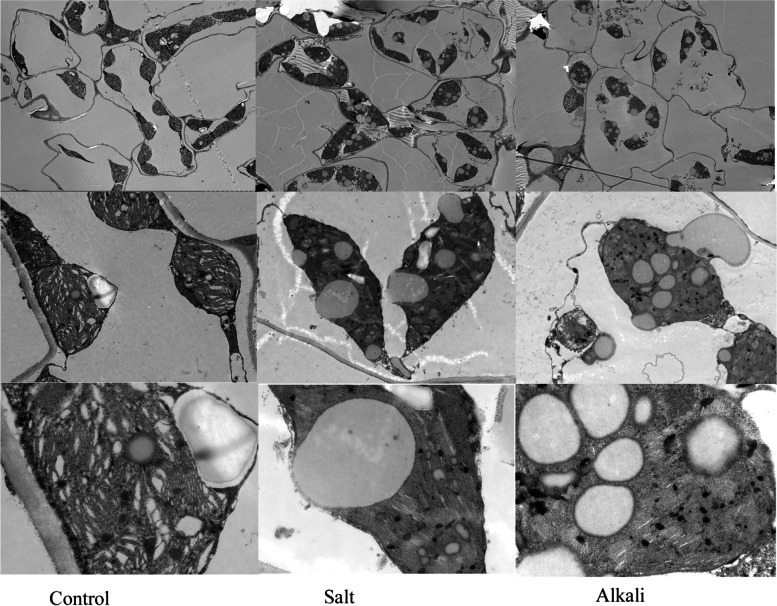
The chloroplast ultrastructure features of grapevine leaves shown by TEM. The three figures in the first, second, and third columns show the chloroplast ultrastructure features in grapevine leaves under control (CK), salt (NaCl), and alkali (NaHCO_3_) treatment, respectively. The figure in the first, second, and third rows, were observed at 1000 (Scale bar = 10.0 μm), 5000 (Scale bar = 2.0 μm), and 15,000 (Scale bar = 1.0 μm) fold magnifications, respectively

The inhibition of plant photosynthesis in plants under salt stress is closely related to the damage to chloroplast structure. In the control plants, the chloroplast structure was oval and arranged orderly (Fig. 2). The chloroplast expanded and rounded, and morphology was changed in salt-treated or alkali-treated plants. In addition, the number of chloroplasts decreased significantly, and the grana lamellae stacked and blurred following salt stress and alkali stress compared to control plants. Particularly, the number of plastid globules and osmiophilic granules was increased, and the volume became larger under two salt stresses compared to control plants. Furthermore, the rupture of the chloroplast envelope was observed.

Salt stress and alkali stress significantly affected leaf phenotype, physiological parameters, and chloroplast ultrastructure. To gain insight into how plants respond to salt stress and alkali stress at the gene level, transcriptome sequencing was performed.

### Overall transcriptome data

According to the experimental design, nine samples were sequenced and mapped to the reference transcriptome. A total of 79.09 Gb clean data were obtained. The effective data volume of each sample was 7.02 Gb, the Q30 scores with each sample were not less than 90.30%, and the average GC content was 46.1% (Table 1).

**Table 1:** sequencing data statistics

Samples	Clean reads	Clean bases	GC Content %	% ≥ Q30
CK-1	31,219,163	9,303,232,408	45.87	91.95
CK-2	30,415,501	9,059,872,846	46.18	90.30
CK-3	30,561,093	9,089,899,090	46.00	91.76
T1-1	30,483,684	9,075,524,030	46.59	92.31
T1-2	29,632,188	8,794,753,438	46.35	92.07
T1-3	29,890,213	8,890,481,606	46.46	91.74
T2-1	32,046,493	9,532,552,320	45.82	92.89
T2-2	27,922,709	8,324,199,288	45.74	92.18
T2-3	23,561,766	7,020,030,120	45.89	90.99

**Fig. 3:**
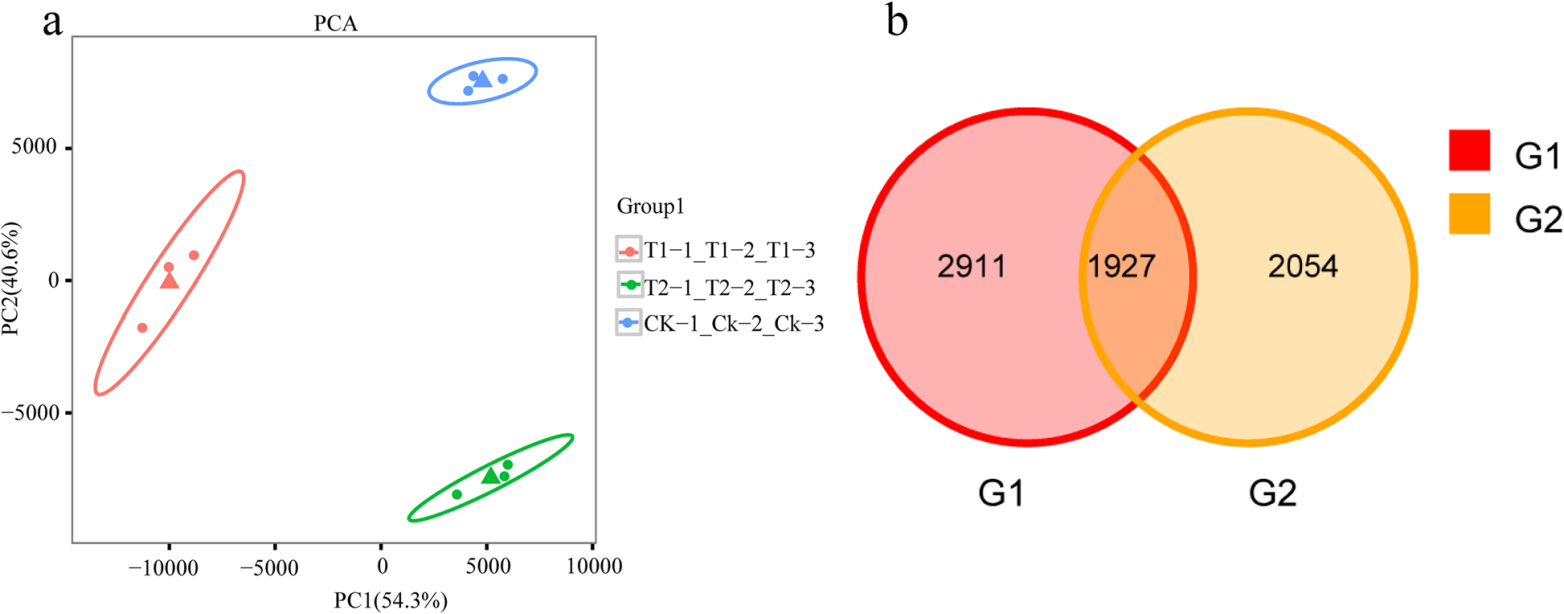
The principal component analysis (PCA) plot and Venn Diagram for DEGs. **a** The PCA analysis for DEGs in grapevine plants treated with control(CK), NaCl (T1 treatment), and NaHCO_3_ (T2 treatment). **b** The Venn diagram of DEGs in the comparisons of salt stress with control (G1 group) and the comparisons of alkali stress with control (G2 group)

A high similarity among the three biological replicates within each treatment was revealed by the principal component analysis (PCA) plot of the transcriptomic data. Meanwhile, there is a clear separation between salt-treated, alkali-treated, and control samples in grapevine plants (Fig. 3a). Statistical results showed that 4838 and 3981 DEGs were identified in the comparisons of salt stress with control (G1 group) and the comparisons of alkali stress with control (G2 group)., respectively. Of the DEGs in the G1 group, 1927 DEGs were common with DEGs in the G2 group, and salt stress and alkali stress induced uniquely 2911 and 2054 DEGs, respectively (Fig. 3b).

### Analysis of GO enrichment for DEGs

**Fig. 4:**
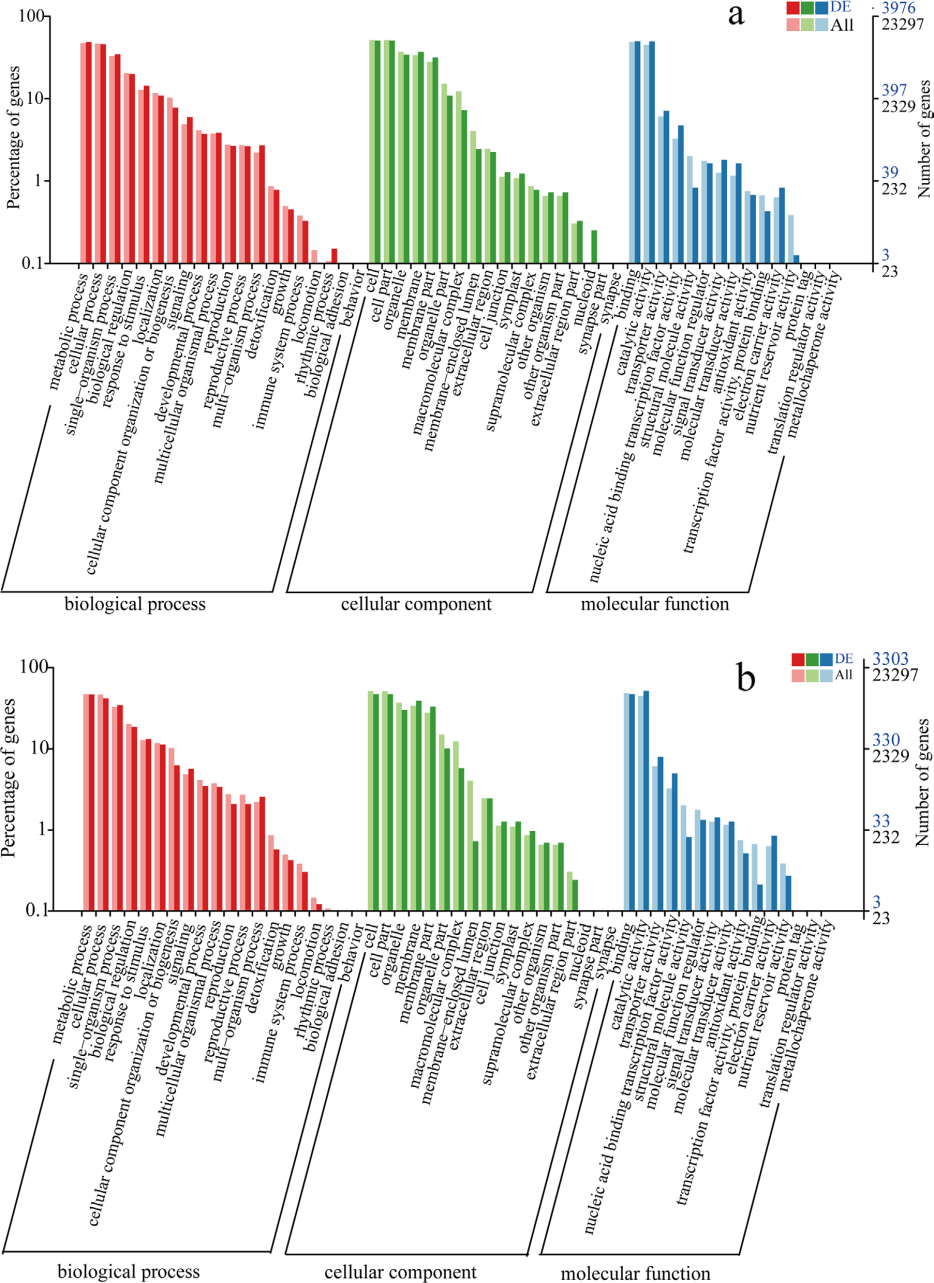
The GO classification for DEGs. **a**, **b** GO classification for DEGs in the G1 group and G2 group, respectively

A GO enrichment analysis of these DEGs was performed to investigate the biological functions of DEGs in grapevine plants under salt stress and alkali stress. GO classification showed that many DEGs from the G1 group (Fig. 4a) and G2 group (Fig. 4b) were enriched into ‘metabolic process,’ ‘cellular process,’ and ‘single-organism process’ in biological process, ‘cell,’ ‘cell part,’ ‘organelle,’ ‘membrane,’ and ‘membrane part’ in a cellular component, and ‘binding’ and ‘catalytic activity’ in molecular function. The GO enrichment indicate that these salt stress and alkali stress-induced DEGs participate in multiple biological processes and have varied molecular functions.

### Analysis of KEGG enrichment for DEGs

**Fig. 5:**
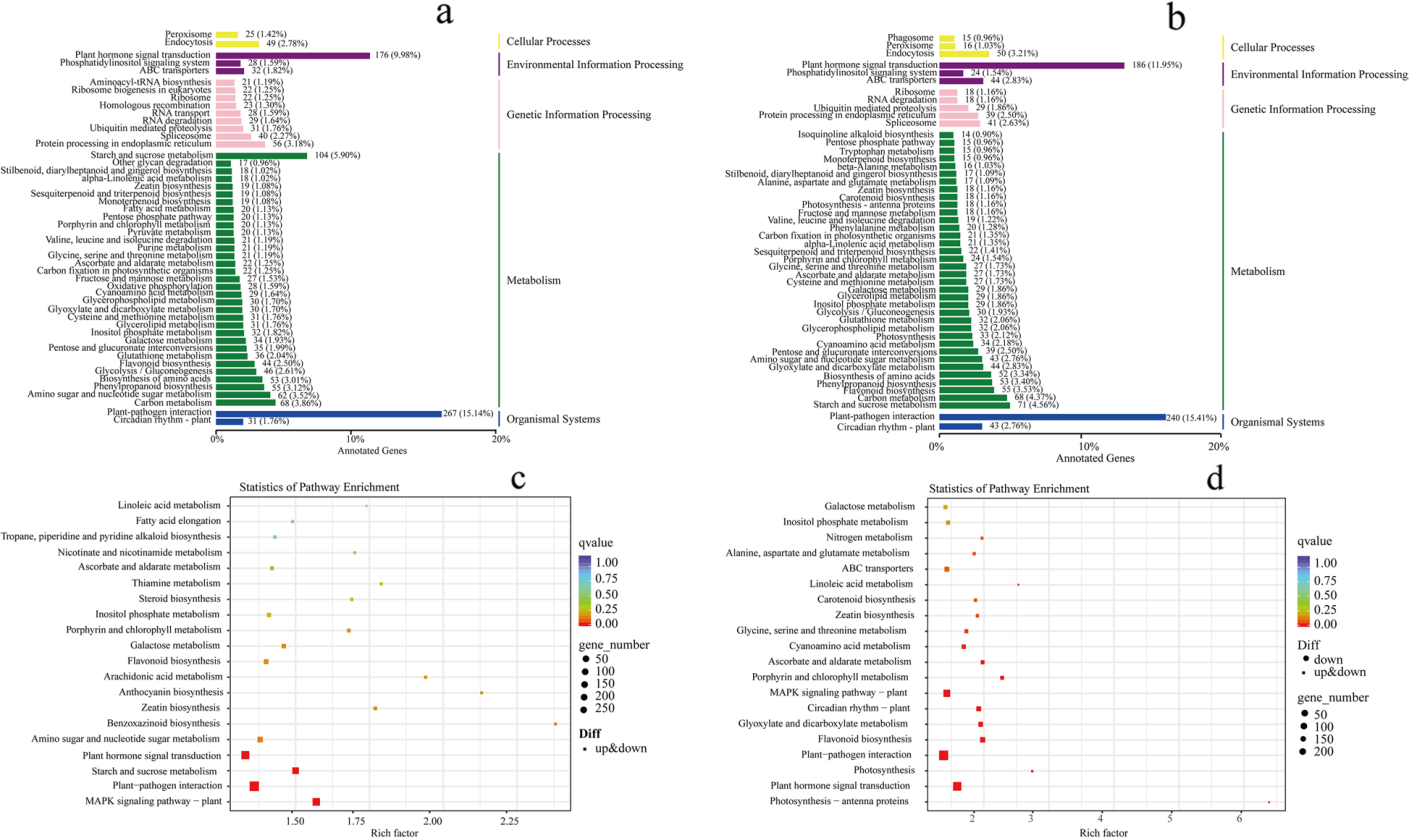
The KEGG classification and the top 20 pathways. **a**, **b** KEGG classification for DEGs in G1 group and G2 group, respectively. **c**, **d** the top 20 pathways in G1 group and G2 group, respectively

To investigate key biological pathways of grapevine plants in response to salt and alkali stress, KEGG enrichment analysis was performed on these DEGs from the G1 group and G2 group. KEGG classification showed that DEGs from the G1 group (Fig. 5a) and G2 group (Fig. 5b) were enriched in ‘Cellular Processes,’ ‘Environmental Information Processing,’ ‘Genetic Information Processing,’ ‘Metabolism,’ and ‘Organismal Systems’ categories, respectively. Of these 20 pathways from the G1 group (Fig. 5c) and G2 group (Fig. 5d), 10 pathways were commonly enriched into the G1 group and G2 group, including ‘Plant hormone signal transduction,’ ‘MAPK signaling pathway-plant,’ ‘Linoleic acid metabolism,’ ‘Ascorbate and aldarate metabolism,’ ‘Inositol phosphate metabolism,’ ‘Porphyrin and chlorophyll metabolism,’ ‘Galactose metabolism,’ Flavonoid biosynthesis,’ ‘Zeatin biosynthesis,’ and ‘Plant-pathogen interaction.’ However, 10 pathways in the G1 group were uniquely enriched, including ‘Fatty acid elongation,’ ‘Tropane, piperidine and pyridine alkaloid biosynthesis,’ ‘Nicotinate and nicotinamide metabolism,’ ‘Thiamine metabolism,’ ‘Steroid biosynthesis,’ ‘Arachidonic acid metabolism,’ ‘Anthocyanin biosynthesis,’ ‘Benzoxazinoid biosynthesis,’ ‘Amino sugar and nucleotide sugar metabolism,’ and ‘Starch and sucrose metabolism.’

In the G2 group, 10 pathways were uniquely enriched, including ‘Nitrogen metabolism,’ ‘Alanine, aspartate and glutamate metabolism,’ ‘ABC transporters,’ ‘Carotenoid biosynthesis,’ ‘Glycine, serine and threonine metabolism,’ ‘Cyanoamino acid metabolism,’ ‘Circadian rhythm-plant,’ ‘Glyoxylate and dicarboxylate metabolism,’ ‘Photosynthesis,’ and ‘Photosynthesis-antenna proteins.’ KEGG enrichment showed that the response of plants to saline and alkali stress was mainly achieved by regulating gene expression involved in signaling transduction and many metabolic pathways.

Next, we investigated the expression of genes involved in some important pathways based on physiological measurement, GO and KEGG enrichment to identify the key genes that plants activate in response to salt and alkali stress.

### The DEGs related to signal transduction

**Fig. 6:**
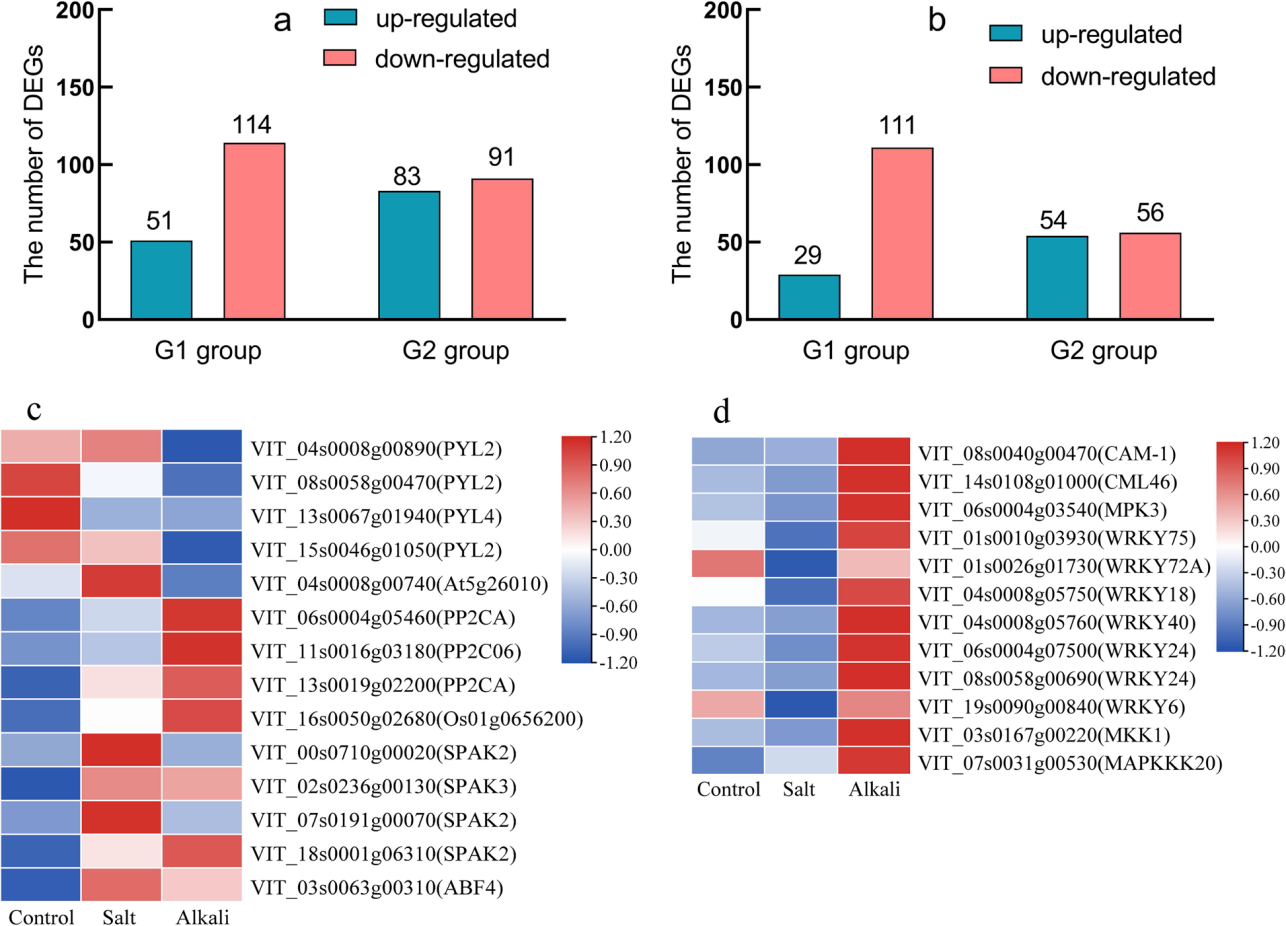
The number of DEGs enriched in the signal pathways and the expression patterns of DEGs. **a** The number of DEGs in ‘Plant hormone signal transduction’ pathway. **b** The number of DEGs in ‘MAPK signaling pathway-plant’ pathway. **c** Heatmap showed the expression patterns of DEGs related to ABA signal transduction. **d** Heatmap showed the expression patterns of DEGs related to MAPK signal transduction

In the ‘Plant hormone signal transduction’ pathway, 165 and 174 DEGs were significantly enriched in G1 group and G2 group (Fig. 6a, Table S2), respectively. In G1 group, there were 51 up- and 114 down-regulated genes. In G2 group, there were 83 up- and 91 down-regulated genes. In the ‘MAPK signaling pathway-plant’ pathway, we identified 140 and 110 DEGs in G1 group and G2 group (Fig. 6b, Table S3), respectively. In the G1 group, 29 up- and 111 down-regulated genes were identified. In the G2 group, 54 up- and 56 down-regulated genes were identified. In the ABA signal pathway, 14 DEGs encoding the PYR/PYL, PP2C, SnRK2, and ABF proteins were identified in the G1 and G2 groups (Fig. 6c). Many genes related to salt tolerance of plants were observed, including *CAM4*, *MPK3*, *WRKY33*, *MKK1*, and *MAPKKK20* (Fig. 6d).

### The DEGs related to Na^+^ and K^+^ transport

**Fig. 7:**
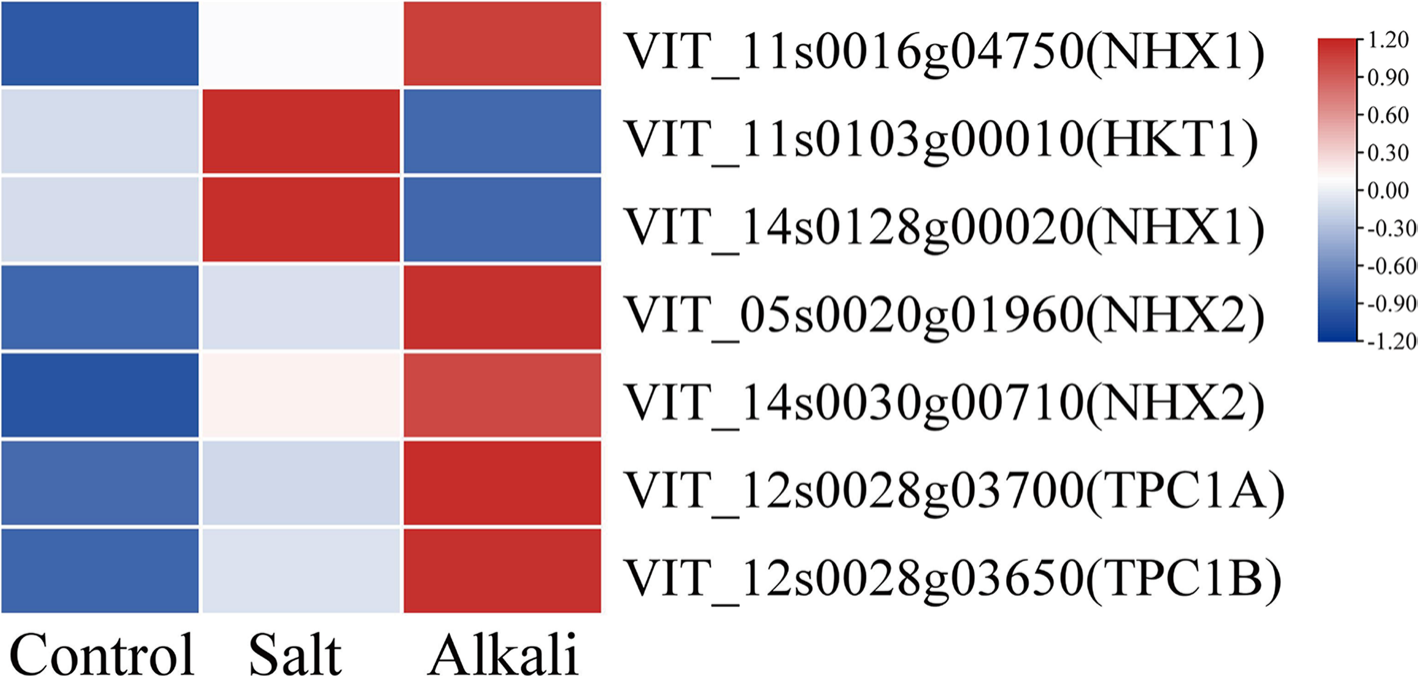
The expression patterns of DEGs related to Na^+^ and K^+^ transport

We identified six DEGs related to ion transport in plant cells (Fig. 7, Table S4), including *AKT1*, *HKT1*, *NHX1*, *NHX2*, *TPC1A*, and *TPC1B*. Among these genes, the *AKT1* and *HKT1* expression levels were lower than those of the other genes. In the G1 group, *AKT1*, *HKT1* and *NHX1* were up-regulated. In the G2 group, *AKT1*, two *NHX2*, *TPC1A*, and *TPC1B* were significantly up-regulated.

### The DEGs involved in ‘Porphyrin and chlorophyll metabolism’ and ‘Photosynthesis-antenna proteins’ pathway

**Fig. 8:**
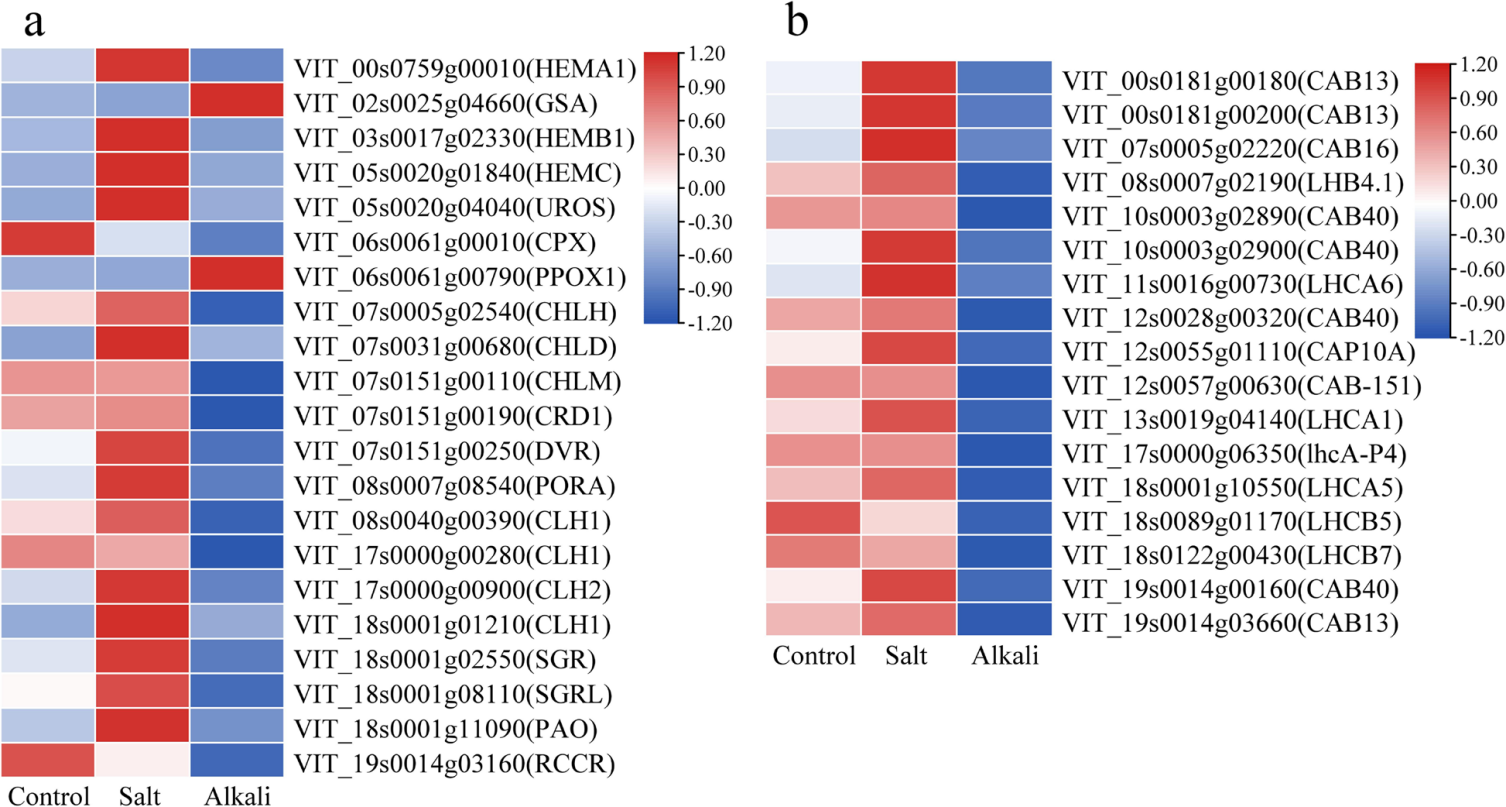
The expression patterns of DEGs related to ‘Porphyrin and chlorophyll metabolism’ pathway (**a**) and ‘Photosynthesis-antenna proteins’ pathway (**b**)

The ‘Porphyrin and chlorophyll metabolism’ pathway was related to chlorophyll metabolism in plants. In this pathway, 12 up- and two down-regulated DEGs in the G1 group and two up- and 11 down-regulated DEGs in the G2 group were related to chlorophyll synthesis and degradation (Fig. 8a. Table S5). Of these DEGs involved in chlorophyll synthesis, seven of eight in the G1 group were up-regulated, but all eight in the G2 group were down-regulated. Of these DEGs related to chlorophyll degradation, there were four up- and one down-regulated DEGs in the G1 group and three up- and two down-regulated DEGs in the G2 group. In the ‘Photosynthesis-antenna proteins’ pathway, 17 DEGs were annotated as chlorophyll a and b binding protein’ (Fig. 8b, Table S6). In the G1 group, five DEGs were up-regulated, and the remaining 12 genes showed no differential expression. However, all 17 genes in the G2 group exhibited down-regulation.

### The DEGs involved in ‘Photosynthesis’ and ‘Carbon fixation in photosynthetic organisms’ pathway

**Fig. 9:**
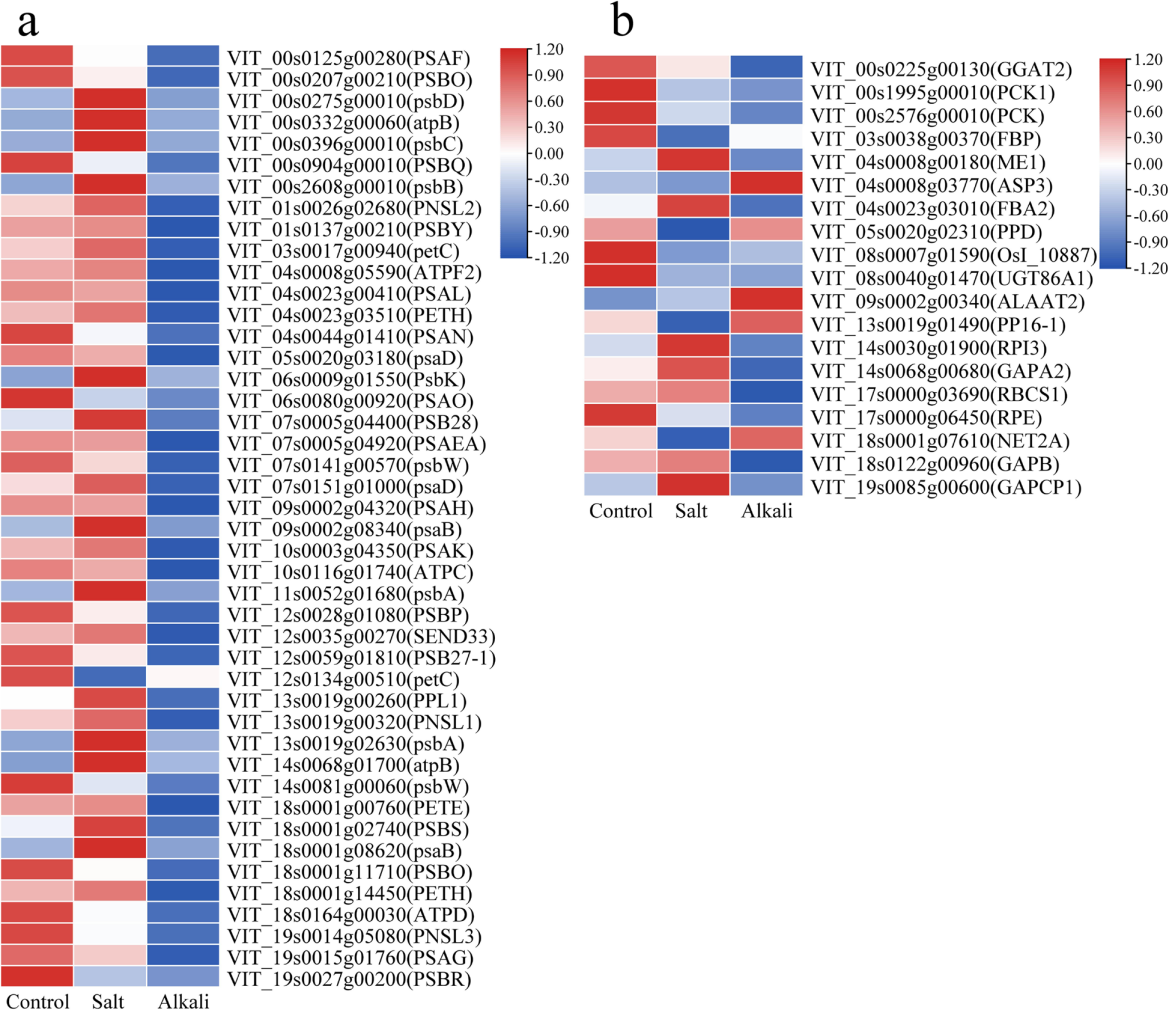
The expression patterns of DEGs related to the ‘Photosynthesis’ pathway (**a**) and ‘Carbon fixation in photosynthetic organisms’ pathway (**b**)

In the ‘photosynthesis’ pathway, DEGs encoded proteins responsible for such activities as electron transport, water-splitting, subunits of photosystem I or II, and ATPase (Fig. 9a, Table S7). In the G1 group, 11 out of 15 DEGs were up-regulated under salt stress. In the G2 group, all 33 DEGs encodings were down-regulated under alkali stress. In the ‘Carbon fixation in photosynthetic organisms’ pathway (Fig. 9b, Table S8), there were three up- and nine down-regulated DEGs in the G1 group. In the G2 group, there were two up- and 11 down-regulated DEGs. An *RBCS1* (Ribulose bisphosphate carboxylase small chain) gene encoding Rubp, was down-regulated under alkali stress compared to control but was not differentially expressed under salt stress.

### Validation of RNA-Seq analysis using quantitative RT-PCR

**Fig. 10:**
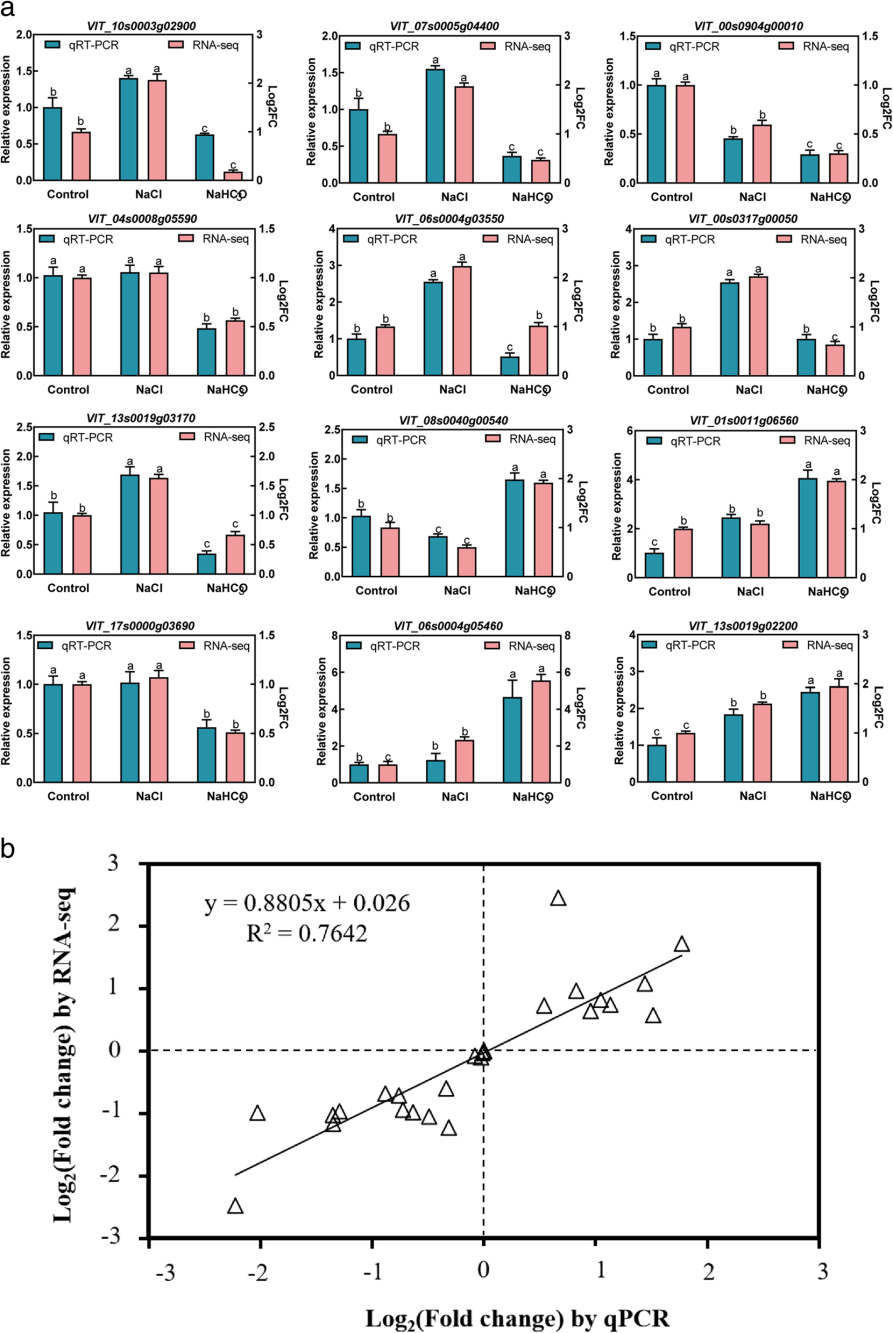
The correlation between qPCR and RNA-seq

To validate the reliability of RNA sequencing data, 12 DEGs were randomly selected to perform qRT-PCR analysis (Fig. 10a). The results showed a high correlation coefficient (R2 = 0.7642) between RNA sequencing data and qRT-PCR results, indicating that the RNA sequencing data are reliable (Fig. 10b).

### Metabolomic analysis

Qualitative and quantitative metabolomic analysis was performed on nine samples. A total of 922 peaks were detected, of which 922 metabolites were annotated. PCA of metabolic data indicated a high similarity among the three biological replicates within each treatment (Figure s1). Moreover, there was a clear separation between the salt-treated, alkali-treated, and control samples.

**Fig. 11:**
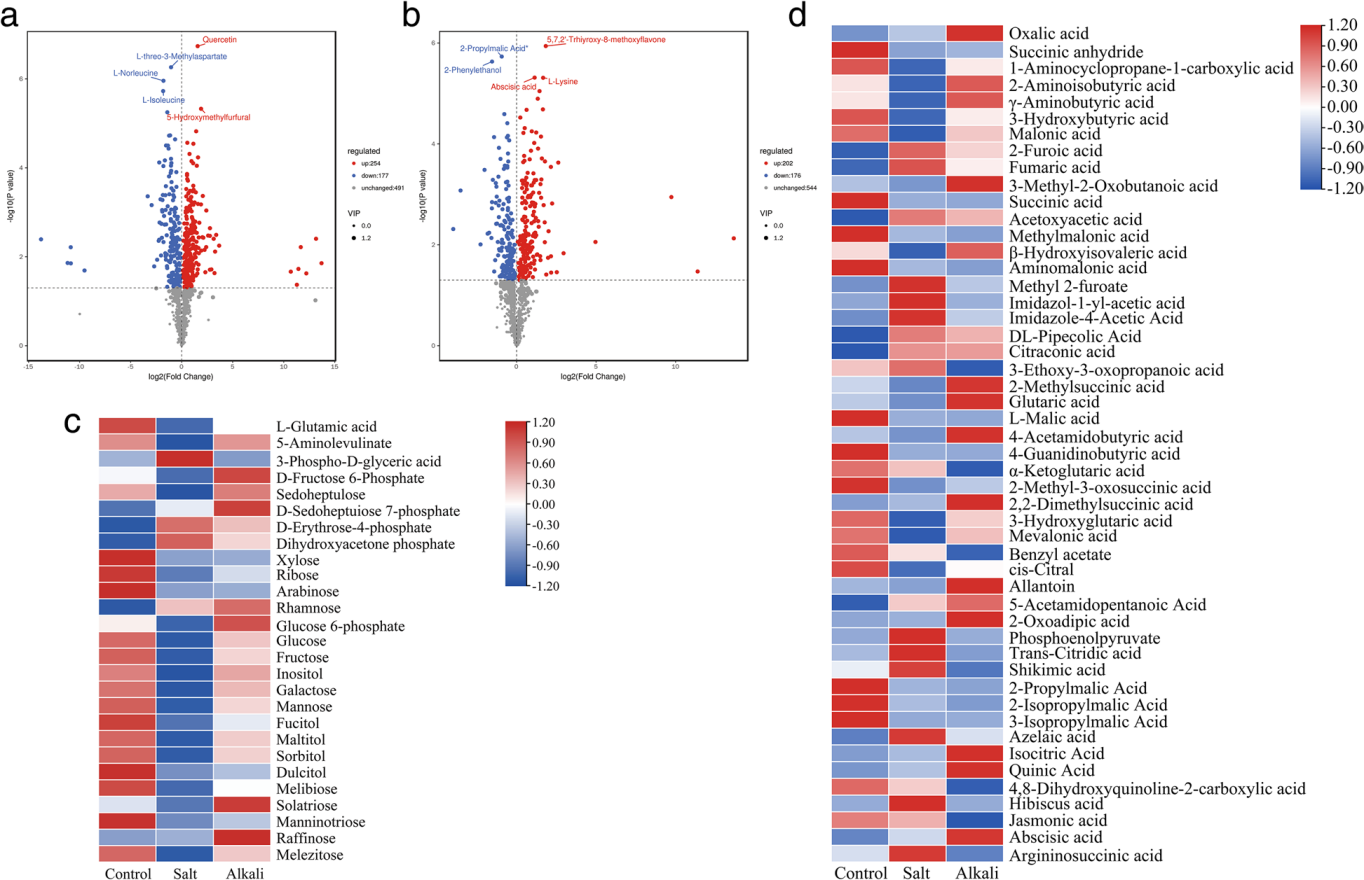
Volcano map for DAMs and the change of DAMs abundance. **a**, **b** Volcano map of DAMs in response to salt stress and alkali stress, respectively. **c** The abundance of DAMs related to ‘chlorophyll synthesis’ and ‘Photosynthesis’. **d** The abundance of organic acids under control, salt stress, and alkali stress

There were 431 and 378 DAMs in the G1 group (Fig. 11a) and G2 group (Fig. 11b), respectively. Analysis of physiological characteristics and transcripts data showed that salt stress and alkali stress significantly affected the gene expression related to chlorophyll metabolism and photosynthesis. Thus, we analyzed DAMs abundance to explore the effect of two salt stresses affecting these biological processes at the metabolic level (Fig. 11c, Table S9). In the ‘Porphyrin and chlorophyll metabolism’ pathway, two substrates involved in chlorophyll synthesis were identified, including L-Glutamic acid and 5-Aminolevulinate (ALA). L-Glutamic acid and 5-Aminolevulinate were enriched in the G1 group, and they were down-regulated; L-Glutamic acid was enriched in the G2 group and down-regulated.

In the ‘Carbon fixation in photosynthetic organisms (ko00710)’ pathway, we identified six metabolites involved in the Calvin cycle pathway. In the G1 group, the abundance of 3-Phospho-D-glyceric acid, D-Sedoheptulose 7-phosphate, D-Erythrose-4-phosphate, and Dihydroxyacetone phosphate increased, but D-Fructose 6-Phosphate and Sedoheptulose decreased. In the G2 group, in addition to 3-Phospho-D-glyceric acid, the abundance of D-Fructose 6-Phosphate, Sedoheptulose, D-Sedoheptulose 7-phosphate, D-Erythrose-4-phosphate, and Dihydroxyacetone phosphate increased.

Among 22 sugar metabolites (sugar and alcohol), In the G1 group, the abundance of two DAMs decreased (Rhamnose and Sedoheptulose 7-phosphate), one DAM was unchanged (Raffinose), and the remaining 19 DAMs decreased. In the G2 group, the abundance of seven DAMs increased (Rhamnose, Sedoheptulose, Sedoheptulose 7-phosphate, Fructose 6-Phosphate, Glucose 6-phosphate, Solatriose, and Raffinose), seven DAMs decreased (Xylose, Ribose, Arabinose, Glucose, Fucitol, Maltitol, and Manninotriose), and eight DAMs were unchanged (Fructose, Inositol, Galactose, Mannose, Sorbitol, Dulcitol, Melibiose, and Melezitose).

Among organic acids (Fig. 11d, Table S10), the abundance of 16 out of 38 DAMs under salt stress increased compared with control, and 20 out of 40 DAMs increased under alkali stress. The abundances of oxalic acid, 2-furoic acid, fumaric acid, acetoxyacetic acid, DL pipecolic acid, citraconic acid, azelaic acid, and abscisic acid in leaves under salt stress and alkali stress were significantly higher than those in the control. Moreover, the abundances of oxalic acid, 2-Aminoisobutyric acid, γ-Aminobutyric acid, 3-Methyl-2-Oxobutanoic acid, glutaric acid, 4-Acetamidobutyric acid, 2,2-Dimethylsuccinic acid, allantoin, 2-Oxoadipic acid, quinic acid, and abscisic acid in leaves under alkali stress were higher than those salt stress.

## Discussion

Grapevine plants adapted to arid and semi-arid environments are affected by salt stress and alkali stress [25, 26]. However, salt stress and alkali stress are two different stresses. In this study, we investigated the effect of salt and alkali stress through physiological determination, transmission electron microscopy, and transcriptome and metabolome analyses to examine the effects of salt and alkali stress on grapevine plants and the differences in grapevine plants between two distinct stresses.

### The genes in the signalling pathways response to salt stress and alkali stress

ABA (abscisic acid), a central regulator of many plant responses to environmental stresses, plays an irreplaceable role in salt stress defense [31]. Genes involved in the ABA signaling pathway are critical for plant stress resistance. In *Sophora alopecuroides* under salt and alkali treatment, five genes (*SaPYL4-1*, *SaPYL4-2*, *SaPYL4-3*, *SaPYL4-4*, and *SaPYL5-1*) encoding PYL protein were down-regulated [32]. Similarly, our results showed that two *PYL4* genes induced by salt stress and three genes (two *PYL4* genes, one *PYL9* gene) induced by alkali stress showed down-regulation. The up-regulation of the *PP2C* gene [9] and overexpression of *PtSnRK2.5* and *PtSnRK2.7* [10] enhance salt tolerance of *Arabidopsis* plants. In sorghum plants, an obvious up-regulation of these genes (*SbPP2C09*, *SbPP2C23*, *SbPP2C52*, *SbPP2C54*, *SbPP2C58*, *SbSAPK1*, *SbSAPK5*, and *SbSAPK9*) occurred under saline-alkali stress [33]. Similarly, in this study, multiple genes genes encoding PP2C and SnRK2 were also up-regulated in grapevine plants under salt stress and alkali stress. Furthermore, high salinity stresses induced the up-regulation of *AREB1/ABF2*, *AREB2/ABF4*, and *ABF3/DPBD5* in *Arabidopsis* [34]. In this study, an *ABF4* gene was up-regulated under salt stress and alkali stress. The above results indicated that grapevine plants altered the expression of key stress genes involved in the ABA signaling pathway, enabling plants to adapt to the stress environment.

In the ‘MAPK signaling pathway-plant’ pathway, multiple genes (such as *CAM4*, *MPK3*, *WRKY33*, *MKK1*, and *MAPKKK20*) were related to the salt tolerance of plants*.* In this study, these genes were up-regulated under alkali stress; conversely, in addition to the *MAPKKK20* gene, the remainder were down-regulated under salt stress*.* Improving *MsCML46*, *MPK3*, *MKK1*, and *WRKY33* expression enhanced the salt tolerance of tobacco [11], potatoes [12], *Arabidopsis* [35, 36]. In *Arabidopsis*, there was a greater accumulation of superoxide occurring in *mkkk20* mutants, but transgenic plants overexpressing *MKKK20* exhibited tolerance to salt stress [37]. Notably, the *MAPKKK20* gene was up-regulated under both salt stress and alkali stress in this study, and a significantly higher expression was observed under alkali stress relative to salt stress. These results indicate that these genes respond more strongly to alkaline stress than salt stress and thus may be important contributors to alkali tolerance in grapevine plants.

### Na^+^ and K^+^ homeostasis in grapevine leaves under salt stress and alkali stress

In this experiment, grapevine plants were exposed to salt stress and alkali stress for a long time leading to chlorosis of the leaf. Also, plants had more chlorotic leaves under alkali stress than salt stress. Chlorotic leaves are often induced by the excessive accumulation of Na^+^ [38] and lack of K^+^ [39] in leaves caused by salt stress and alkali stress. In this study, the changes in Na^+^ and K^+^ level under alkali stress were significantly greater than those under salt stress, similar to results reported for apple [40], tomato [41], and wheat [42]. Therefore, our findings and previous studies have indicated that relatively more Na^+^ and less K^+^ were accumulated in plants under alkali stress than under salt stress. This may be because the high pH induced by alkali stress destroys the root membrane, resulting in plants absorbing more sodium ions than salt stress. At the same time, potassium ions under alkali stress are lower than that under salt stress due to the antagonism between sodium and potassium ions [43].

The above results indicated that salt stress and alkali stress increased Na^+^ and decreased K^+^ in grapevine leaves, disrupting the ion imbalance and decreasing the K^+^/Na^+^ ratio. To balance the toxic effect of Na^+^ accumulation and maintain high cytoplasmic K^+^/Na^+^ ratios during salinity stress, plants needed to maintain stable K^+^ acquisition and distribution by controlling the expression of gene encoding ion transporter. Studies shows that the overexpression of *NHX1* and *NHX2* genes could improve the salt tolerance of many plant species, such as *Arabidopsis* [44], wheat [45], tomato [46]. In our experiment, we found that salt stress enhanced the *NHX1* gene expression, but alkali stress improved *NHX2* gene expression.

The AKT1 protein participates in high-affinity K^+^ absorption to ensure a constant K^+^ supply, resulting in a high internal-K^+^ to external-K^+^ concentration [47, 48]. In *Arabidopsis*, the overexpression of *PutAKT1* increased K^+^ uptake by cells and reduced Na^+^ accumulation [49]. In this study, the results showed that both salt stress and alkali stress induced the up-regulation of the *AKT1* gene in grapevine leaves. Similar results were also found in soybean leaves and roots [50]. *AtHKT1* encodes a sodium (Na^+^) transporter and plays an important role in regulating salt stress tolerance in plants. It is generally accepted that Na^+^ can be retransmitted to lower leaves and roots through HKT1 protein to prevent excessive accumulation in photosynthetic tissues [51, 52]. Salt stress could induce the expression of *AtHKT1;1* in *Arabidopsis*, reducing the Na^+^ content in plants and alleviating toxicity [53]. Similar to the studies in *Arabidopsis*, our results indicated that the *HKT1* gene was up-regulated under salt stress. Salt stress-induced long-distance Ca^2+^ wave is dependent on the activity of the vacuolar cation channel protein two-pore channel 1 (TPC1) channel protein, which appears to contribute to whole-plant stress tolerance. In *Arabidopsis*, TPC1 plays an important role in cation homeostasis and vacuolar storage, mediating the passage of Na^+^ and K^+^ [54]. This study showed that two *TPC1* (*TPC1A* and *TPCB*) genes showed up-regulation under alkali stress.

Remarkably, the researchers found the weakening of stomatal conductance was closely related to the imbalance of Na^+^-K^+^ in plant cells. Some evidence has confirmed that the AKT1, NHX1, and NHX2 ion transporter proteins regulate plants’ stomatal movement. Silencing of the *AKT1* gene in *Arabidopsis thaliana* damaged the K^+^ transmembrane transport system of guard cells and decreased stomatal conductance[55]. The *NHX1* and *NHX2* genes are greatly expressed in guard cells of stomata, where they contribute to the regulation of stomatal function and transpiration [56, 57]. Similar to Nieves-Cordones et al. [55], Barragán proposed [56] that dysfunctional stomatal regulation in *nhx1 nhx2* mutant plants is presumably linked to the reduced ability to compartmentalize K^+^ in the vacuoles of guard cells. In this study, we identified two up-regulated genes [*AKT1* and *NHX2* (VIT_14s0030g00710)] enriched in ‘Regulation of stomatal closure (GO:0,090,333).’ The above results indicate that grapevine plants, during exploring salt stress and alkali stress, may regulate K^+^ transport in plant cells by enhancing the expression of *AKT1* and *NHX2* (VIT_14s0030g00710) genes in grapevine leaves to achieve the purpose of regulating stomatal movement.

Additionally, organic acids secreted in plants under saline-alkali stress could neutralize excess cations, such as Na^+^_,_ to maintain charge balance. In tomato[41] seedlings treated with salt or alkali stress, accumulation of organic acids, such as oxalic acid and citric acid, was observed. In this study, 16 and 22 organic acids with increased abundance were identified in grapevine leaves under salt stress and alkali stress, respectively. The above results suggest that grapevine plants during salt stress and alkali stress may up-regulate *AKT1*, *HKT1*, *TPC1A*, *TPC1B*, *NHX1*, and *NHX2*, and increase organic acids in response to Na ^+^ and K ^+^ homeostatic imbalance.

### Salt stress and alkali stress decreased photosynthesis efficiency in grapevine plants by stomatal and non-stomatal factors

Photosynthesis was negatively affected during high salinity exposure. In this experiment, we investigated the gas exchange parameters (Pn, Gs, Ci, Tr) to evaluate the effect of two salt stresses on photosynthesis. The results showed that the four indexes decreased under salt stress and alkali stress. A similar phenomenon occurred in grapevine [27, 28], apple [40], tomato [41], and wheat [42]. In this study, the obvious decrease of stomatal conductance was identified in grapevine plants under two salt stresses, suggesting the diffusion of CO_2_ from the environment to the chloroplast was blocked under salt stress and alkali stress and reduced the photosynthetic carbon assimilation [58]. It was considered that the decrease in Pn was due to either reduced intracellular CO_2_ partial pressure caused by stomatal closure or non-stomatal factors. The non-stomatal factors included the reduction of K^+^ in plant cells caused by the accumulation of Na^+^, decrease in photosynthetic pigments, damage of chloroplast ultrastructure, and decrease in key photosynthetic enzyme activity. In this study, we observed a reduction of chlorophyll content, a decrease in Rubisco activity, and damage of chloroplast ultrastructure in grapevine plants under salt stress. Thus, the above results indicated that two salt stresses decreased photosynthesis efficiency in grapevine plants by stomatal and non-stomatal factors. Significantly, we found that the bad effect of alkali stresses on gas exchange indices was more marked than salt stress. Similar results were also reported in previous studies [40–42]. This may be due to the high pH value and higher osmotic stress caused by alkali stress [59].

Light energy captured by the photosynthetic apparatus is mainly used for photosynthesis, but a small portion is dissipated in the form of fluorescence and heat energy. The chlorophyll fluorescence index Fv/Fm is used as a photoinhibition index that reflects the efficiency of light energy conversion in the active center of PS II [60]. In this study, there are obvious differences in Fv/Fm between the control and two stress treatments. We also observed that Fv/Fm was lower under alkali stress than under salt stress. A similar finding also was reported in Chinese cabbage [61]. These results indicate that salt stress and alkali stress causes impairment of photochemical conversion efficiency in PSII protein complexes, and alkali stress has a more bad effect on the active center of PS II than salt stress. NPQ played a key role in dissipating excess light energy [62, 63]. The increase of NPQ has been thought to be an energy dissipation mechanism that protects the photosynthetic system [63, 64]. However, in this study, NPQ was higher under salt stress than in the control plants, but this trend was reversed under alkali stress, similar to findings in apple [40]. These results indicate that grapevine plants can resist salt stress by triggering heat dissipation protection under salt stress, while the heat dissipation self-protection ability in plants is weakened under alkaline stress.

Photosystems PSI and PSII play a crucial role in the absorption and conversion of light energy, photosynthetic electron transport, and photosynthetic carbon fixation [65]. Previous studies have shown that salt stress led to the down-regulation of many PSI and PSII genes in sweet sorghum [66], wild Jujube [67], and *Fraxinus velutina* Torr [68]. In this study, all 17 genes enriched in the ‘Photosynthesis-antenna proteins’ pathway were down-regulated under alkali stress, indicating grapevine plants might attempt to decrease the absorbance of light energy by reducing the light-harvesting complexes to protect grapevine plants in an alkali stress environment [69]. Moreover, 33 DEGs enriched in the ‘photosynthesis’ pathway were down-regulated under alkali stress. This suggests that alkali stress could suppress the activity of PSI and PSII, reducing photosynthetic efficiency [69]. In contrast, our results showed that the genes enriched in the ‘Photosynthesis-antenna proteins’ showed no differential expression under salt stress, and the genes enriched in the ‘photosynthesis’ were up-regulated. Therefore, we propose that the negative effect of alkaline stress on the PSI and PSII genes is more severe than that of salt stress at the transcriptional level, thus leading the lower photosynthetic efficiency in alkali stress-treated plants than that of salt stress-treated plants.

Notably, of these genes in the ‘photosynthesis’ pathway in this study, a *PSBS* gene was identified. The *PSBS* gene encodes the PSBS protein, which plays an important role in non-photochemical quenching [70]. Higher NPQ in lines of overexpression *PsbS* (L17) was observed, but lower NPQ and remarkable reduction of electron transport rate in PsbS-lacking *npq4* mutant was identified compared to wild type [71]. The *npq4* mutant (lacking PsbS protein) showed a greater extent of photosystem II photoinhibition due to more singlet oxygen (^1^O_2_) in chloroplasts [70]. In this study, the expression of the *PSBS* (VIT_18s0001g02740) gene was increased 1.45-fold under salt stress relative to control. However, the expression *PSBS* gene under alkali stress was significantly decreased relative to the control. The trend of this *PSBS* gene was consistent with NPQ under salt stress and alkali stress. Thus, we propose that grapevine plants’ have improved expression of the *PSBS* gene under salt stress, which may enhance the function of psbs protein in regulating non-fluorescent chemical quenching to dissipate excess light energy. Conversely, the down-regulation of the *PSBS* gene induced by alkali stress may weaken the ability of heat dissipation and lead to chloroplast oxidative stress caused by low electron transfer efficiency.

### Salt stress and alkali stress affected chlorophyll metabolism

In higher plants, chlorophyll plays a key role in light harvesting and energy transduction in photosynthesis. In this study, a reduction of 41.32% and 62.15% in total chlorophyll contents of grapevine leaves was observed under salt stress and alkali stress, respectively, which indicated that alkali stress led to a greater reduction in chlorophyll content compared to salt stress. This finding was in agreement with previous reports [40–42]. The decrease in plant chlorophyll levels under salt stress was attributed to the inhibition of chlorophyll synthesis and the activation of chlorophyll enzymes for its degradation [72].

Chl biosynthesis proceeds through a series of reactions, and the synthesis of Chl is affected if any of these steps are disrupted [73]. It was reported that salt stress and alkali stress have adverse effects on chlorophyll synthesis, which may be caused by the decrease of protein or gene expression in chlorophyll synthesis under the two stress conditions [59]. Here we identified eight down-regulated DEGs related to chlorophyll synthesis in the G2 group (including *HEMA*1, *HEMB1*, *PPOX1*, *CHLH*, *CHLD*, *CHLM*, *CRD1*, *PORA*). Similar to this, the down-regulation of *HEMA1* and *CHLH* genes was observed in cucumber [74] and *Nicotiana benthamiana* seedlings following exposure to salinity stress [75]. Thus, the chlorophyll content in grapevine leaves under alkali stress was lower than that of control and salt stress, which may be due to alkali stress significantly inhibiting the expression of genes related to chlorophyll synthesis. In the G1 group, there were 10 DEGs related to chlorophyll synthesis under salt stress. However, in addition to a down-regulated *PORA* (VIT_19s0014g03160) gene, the remaining nine genes [*GSA* (encoding HEML), *HEMB1*, *HEMC*, *UROS* (*HEMD*), *HEME1*, *CPX* (encoding HEMF), *CHLH*, *DVR*, and *PORA* (VIT_12s0059g00270)] showed an up-regulation. This indicated that the inhibition of alkali stress on chlorophyll synthesis-related genes was stronger than salt stress.

In chlorophyll catabolism, the chlorophyll degradation can be attributed to these enzymes, including chlorophyllase (CLH) [76], magnesium dechelatase (SGR, SGRL) [77], pheophorbide a oxygenase [78] (PAO), and red chlorophyll catabolite reductase (RCCR) [79]. It was reported that the up-regulated expression of *SGR* and *CLH* genes could promote the degradation of Chl [80, 81]. The previous finding indicated that submergence-induced reduction of Chl was associated with the down-regulation of Chl-biosynthetic genes and up-regulation of Chl-degrading genes in perennial ryegrass [82]. In this study, in addition to the *SGR* gene, the *CLH1 and CLH2, SGRL*, and *RCCR* genes were up-regulated under salt stress. Nevertheless, three of four *CLH1* genes were down-regulated, but the *SGR* and *PAO* genes were up-regulated under alkali stress. The up-regulation of the *CLH* gene with the decrease of Chl content was also observed in tomato seedlings under salinity-alkalinity stress [83]. These results suggest that salt stress and alkali stress may increase the expression of genes involved in chlorophyll degradation, thereby reducing the chlorophyll content in grapevine leaves.

The reduction in Chl content was related to the precursor involved in chlorophyll synthesis. In higher plants, 5-Aminolevulinic acid (ALA) is a precursor of plant chlorophyll synthesis derived from glutamate [84]. The decrease of chlorophyll content in severely NaCl-stressed sunflower leaves was mainly due to a decrease in ALA synthesis [72]. The impairment of ALA biosynthesis under chill- and heat-stress conditions led to the inhibition of Chl biosynthesis [85]. In this study, salt stress decreased glutamate and ALA. Similarly, the reduction of glutamate and ALA also was found in sunflower leaves during salt stress [73, 86]. Therefore, these findings indicate that the reduction of Chl in grapevine leaves under salt stress might be attributed to decreasing the ALA precursor.

### Salt stress and alkali stress inhibited the carbon fixation

In this study, salt stress and alkali stress induced many down-regulated genes involved in the ‘Carbon fixation in photosynthetic organisms’ pathway, which may reduce carbon fixed efficiency. Rubisco played a key role in photosynthetic carbon fixation. Previous studies have shown that salt stress inhibits Rubisco activity [87] and induces down-regulation of *RBCS* gene expression [87, 88]. Similarly, our results showed that salt stress and alkali stress decreased Rubisco activity, and this *RBCS* gene was down-regulated under alkali stress. However, we also observed that this RBCS gene showed no differential expression under salt stress, and a similar result was identified in NaCl-treated Serratia liquefaciens plants [89].

Salt stress negatively affected the Calvin cycle in carbon fixation, increasing the abundance of metabolites related to the Calvin cycle pathway. The abundance of sedoheptulose-7-phosphate, D-fructose-1,6-bisphosphate, sedoheptulose, and 3-phospho-D-glyceroyl phosphate in *Cyclocarya paliurus* was increased under salt stress [90]. The metabolites in the regeneration pathway of ribulose-1,5-bisphosphate (ribose-5-phosphate and ribulose-5-phosphate) were highly accumulated in salt-stress sugar beet [91]. In this study, 3-Phospho-D-glyceric acid, D-Sedoheptulose 7-phosphate, D-Erythrose-4-phosphate, and Dihydroxyacetone phosphate had relatively increased abundance under salt stress, and D-Fructose 6-Phosphate, Sedoheptulose, D-Sedoheptulose 7-phosphate, D-Erythrose-4-phosphate, and Dihydroxyacetone phosphate were up-regulated under alkali stress. Therefore, we propose that the accumulation of these metabolites in plants under salt stress and alkali stress may be caused by decreased carbon fixation efficiency due to the down-regulation of gene expression.

Sugar metabolites are the main products of photosynthesis, play a key role in stress perception, and signaling, and form a regulatory center for gene expression mediated by adverse environments, thus ensuring osmoregulation responses, scavenging ROS, and maintaining cellular energy status through carbon partitioning [92]. Previous studies have reported that salt stress could induce the accumulation of sugar metabolites [93]. In this study, the abundance of sugar metabolites showed an obvious difference between salt stress and alkali stress. The abundance of these sugar metabolites was down-regulated under salt stress but up-regulated or unchanged under alkali stress, suggesting that these sugar metabolites could be relevant to the observed difference in plant tolerance to salt stress and alkali stress. In ‘Fengtian’ *Stevia rebaudiana* Bert. (Bertoni), salt stress increased the Rhamnose level and decreased Glucose [94], which was also observed in this experiment. Raffinose not only plays a crucial role as osmoprotectants in plants’ response to cold and drought stresses [95, 96], but also plays an important in protecting the photosynthetic apparatus from oxidative damage under cold stress [97]. In this study, the abundance of raffinose was increased under alkali stress. This implies that enhancing the raffinose level in grapevine leaves may protect photosystem II from damage to alkali stress.

## Conclusion

This study investigated the effect of salt stress and alkali stress on grapevine plants and the differences between salt-stressed and alkali-stressed grapevine plants. The chlorosis of grapevine leaves in alkali stress was more severe than in salt stress. Salt stress and alkali stress induced the differential expression of key stress genes involved in the ABA signaling and MAPK signaling pathways. Salt stress and alkali stress up-regulated expression of gene encoding ion transporter and increased the abundance organic acid in response to an imbalance of Na^+^ and K^+^ in grapevine leaves. Alkali stress had more severe effects on stomatal conductance, chlorophyll content and Rubisco activity, and chloroplast ultrastructure, and induced more down-regulated genes involved in ‘Porphyrin and chlorophyll metabolism,’ ‘Photosynthesis-antenna proteins,’ ‘Photosynthesis,’ and ‘Carbon fixation in photosynthetic organisms’ in grapevine plants compared with salt stress, thus leading to photosynthetic capacity in grapevine plants was lower and accumulating more intermediates related to Calvin cycle under alkali stress than under salt stress. Most sugar metabolites in leaves under alkali stress increased, but almost all sugar metabolites in leaves under salt stress exhibited a decline. These results revealed the general effects of salt stress and alkali stress on phenotype, chloroplast structure, physiological characteristics, gene expression, and metabolites in grapevine plants and indicated the adverse effect of alkali stress on grapevine plants was more serious than that of salt stress.

## Methods

### Plant materials and treatment

Two-year-old cuttings of grapevine cultivar *Cabernet* grapevine (*Vitis vinifera* L.) were used as materials. These cuttings were purchased from ZhiChang Agriculture Company in Shandong Provincial. They were planted in plastic pots (27.5 cm in diameter, 31 cm in height, 15 L) filled with a 2:1 (v/v) mixture of vermiculite:nutrition soil. After 8 weeks, 30 plants growing uniformly were selected and divided randomly into three sets. Each plant was considered a single replicate, with 10 replicates per set. One set was used as an untreated control group, and the other two sets were used as stress treatment groups. Neutral salt stress (T1 treatment) and alkali salt stress (T2 treatment) were simulated by applying 200 mM NaCl (pH 6.94) and 200 mM NaHCO_3_ (pH 8.32) (with an increment of 50 mM per day to the final concentration), respectively. The without NaCl and NaHCO_3_ treatment were considered the control condition (CK). After 20 days, the grapevine leaves were used for measurement analysis for gas exchange parameters, chlorophyll fluorescence, chlorophyll content, TEM, ribulose-bisphosphate carboxylase (Rubisco) activity, Na^+^, and K^+^. Some leave samples were frozen immediately in liquid nitrogen and stored at -80 °C for RNA extraction and RNA sequencing. There were 10 replicates for each treatment. All pots of seedlings were maintained in a greenhouse (25.0 ± 1.5 °C at daytime and 20.5 ± 1.5 °C at nighttime, with the photosynthetic photon flux density of 600 μmol m^−2^ s^−1^).

### Measurement of physiological indices

The third and fourth green leaves [28] at the top of the plant were selected for detecting the gas exchange parameters and chlorophyll fluorescence index. Then, these leaves were collected for the determination of chlorophyll content and ion content. Gas exchange parameters, including Net photosynthetic rate (Pn), stomatal conductance (Gs), Intercellular CO_2_ concentration (Ci), and transpiration rate (Tr), were measured by the Li-6400XT photosynthetic system between 8:30 and 11:30 am. The light intensity was controlled at 800 μmolm^−2^ s^−1^ at 25 ± 1 °C. Chlorophyll fluorescence index, the maximum quantum yield of photosystem II (Fv/Fm), and non-photochemical quenching (NPQ) were measured using a handheld chlorophyll fluorescence instrument (FluorPen FP 100, Eco Tech, Beijing, China).

The chlorophyll content and Rubisco (ribulose-1,5-bisphosphate carboxylase/oxygenase) activity were determined using a commercial kit (Beijing solarbio science & technology co.,ltd.). Na^+^ and K^+^ were extracted from the dried plant leaves in 2/1 (v/v) HNO_3_/HClO_4_ at 80 °C for 48 h, and ion analysis was performed using an ICP-MS (Iris Intrepid II; Thermo Electron Corporation, Franklin, MA, USA).

### Transmission electron microscopy (TEM)

The chloroplast ultrastructure assay was performed by Servicebio Company (Wuhan, China), as described below.

#### Sampling and fixing

The small, cut tissue block was transferred to an EP tube filled with fresh electron microscope fixing solution and the air pumped using a vacuum pump until the block had sunk to the bottom of the tube. The sample was then fixed at room temperature for 2 h, then stored at 4 °C.

#### Post fixation

The sample was fixed with 1% osmic acid prepared with 0.1 M phosphate buffer Pb (pH7.4) at room temperature for 7 h. Then rinsed with 0.1 M phosphoric acid buffer Pb (pH 7.4) three times for 15 min each time.

#### Dehydration at room temperature

The sample was treated with 30%, 50%, 70%, 80%, 95%, 100%, and 100% ethanol, respectively, 1 h each time. Then, the sample was treated with absolute ethanol:acetone (3:1) for 30 min, absolute ethanol:acetone (1:1) for 30 min, absolute ethanol:acetone (1:3) for 30 min, and acetone for 1 h.

#### Permeation embedding

Embedding was using using acetone:812 embedding agent (3:1) at 37 °C for 2–4 h; acetone:812 embedding agent (1:1) permeating at 37 °C for overnight; acetone:812 embedding agent (1:3) 37 °C for 2–4 h; and pure 812 embedding agent treatment at 37 °C for 5–8 h. We then added pure 812 embedding agent to the embedding plate, inserted the sample into the embedding plate, and baked it in the oven at 37℃ overnight.

#### Polymerization

An embedding plate was placed in an oven at 60 °C for polymerization for 48 h, and the resin block was then taken out for standby.

#### Ultra-thin slicing

The resin block was cut to obtain 60–80 nm ultra-thin slices using a thin-slice cutting machine and 150 square copper mesh to salvage.

#### Dyeing

The copper mesh was dyed in 2% uranium acetate saturated alcohol solution for 8 min without light; washed with 70% alcohol three times; cleaned with ultrapure water three times; rinsed with 2.6% lead citrate solution to avoid carbon dioxide dyeing for 8 min; washed with ultrapure water three times and dried slightly with filter paper; then placing the copper mesh slices into the copper mesh box and drying at room temperature overnight.

#### Microscopy

A transmission electron microscope was then used for image acquisition and analysis.

### Transcriptome sequencing

RNA sequencing and metabolomic profiling were performed by Biomarker Technologies Co, LTD. Three biological replicates were involved in RNA-Seq analysis. Total RNA was extracted from grapevine leaf tissues by the Aidlab RN40 Plant RNA kit (Aidlab, Beijing, China) following the manufacturer’s instructions. One microgram of RNA per sample was used for RNA-seq library construction using NEBNext UltraTM RNA Library Prep Kit for Illumina (NEB, USA), following the manufacturer’s recommendations. The cDNA library was enriched, purified, and then sequenced on the Illumina NovaSeq 6000 platform. The clean reads were mapped to the reference genome of *Vitis vinifera* L. (IGGP_12x, NCBI), and the database was from ensemblgenomes.org. Gene expression levels were estimated by fragments per kilobase of transcript per million fragments mapped. Differentially expressed genes (DEGs) were identified with a *P* < 0.05 and∣fold change (FC)∣ > 1.5 as the threshold. The DEGs were submitted to the BMKCloud Bioinformatic Platform http://www.biocloud.net/) for gene ontology (GO) and KEGG pathway enrichment analyses. Gene Ontology (GO) enrichment analysis of the differentially expressed genes (DEGs) was implemented by the GOseq R packages [98]. Statistical enrichment of DEGs in the KEGG pathway [99] was performed using KEGG Orthology Based Annotation System (KOBAS) [100] software.

### Metabolomic profiling

Six biological replicates samples were performed for metabolomic profiling through LC–MS/MS. Samples were analyzed on an Ultra Performance Liquid Chromatography system coupled to mass spectrometry. The raw data collected using MassLynx V4.2 were processed by Progenesis QI software. Combining the difference multiple, the *p*-value and the VIP value of the OPLS-DA model were adopted to screen the differentially accumulated metabolites (DAMs). The screening criteria are ∣FC∣ > 1, *p*-value < 0.05 and VIP ≥ 1.

### Quantitative Real-Time reverse transcription PCR analysis

To validate the reliability of transcriptome sequencing data, 12 DEGs from transcriptome data were randomly selected to perform quantitative real-time (qRT)-PCR analysis. Reverse RNA transcription was performed using FastKing gDNA Dispelling RT SuperMix (Tiangen, Beijing, China). Primers for qPCR were designed using Primer Premier 5.0 software (Table S1). LightCycler® 96 Real time-PCR machine (Roche, Germany) was used for qRT-PCR. The reaction system was 20 μL, containing 10 μL 2 × Talent qPCR PreMix, 2 μL 50 ng/μL cDNA, 0.6 μL forward and reverse primers, and 6.8 μL RNase-Free ddH_2_O. The qRT-PCR reaction procedure was: per-denaturation at 95 °C for 3 min, and then 40 cycles of 95 °C, 5 s; 60 °C, 10 s; 72 °C, 15 s. The relative expression of the 10 genes was calculated using *actin-101* as a reference gene by the 2^−ΔΔCt^ method. Each sample group contained three biological replicates.

### Statistical analysis

The figures and tables were made using Excel 2016 and GraphPad Prism 8.0. The statistical significance analysis of the physiological data was performed using IBM SPSS Statistics 19 software; *p* < 0.05 was considered significant. The figures were edited using PhotoShop CC 2019.

## Supplementary Information

**Additional file 1:** **Figure S1.** The principal component analysis (PCA) plot for DAMs.

**Additional file 2:** **Table S1.** Primer sequences used in qRT-PCR analysis.

**Additional file 3:** **Table S2.** The DEGs involved in the ‘Plant hormone signal transduction’ pathway.

**Additional file 4:** **Table S3.** The DEGs involved in the ‘MAPK signaling pathway-plant’ pathway.

**Additional file 5: Table S4.** The DEGs related to ion transport.

**Additional file 6: Table S5.** The DEGs involved in the ‘Porphyrin and chlorophyll metabolism’ pathway.

**Additional file 7: Table S6.** The DEGs involved in the ‘Photosynthesis-antenna proteins’ pathway.

**Additional file 8: Table S7.** The DEGs involved in the ‘Photosynthesis’ pathway.

**Additional file 9: Table S8.** The DEGs involved in the ‘Carbon fixation in photosynthetic organisms’ pathway.

**Additional file 10: Table S9.** The DAMs involved in Chl biosynthesis and photosynthesis.

**Additional file 11: Table S10.** The DAMs related to organic acids.

## Data Availability

The RNA-seq raw datasets generated during the current study have been deposited in NCBI repository (https://www.ncbi.nlm.nih.gov/bioproject/), and login ID of BioProject was PRJNA809636.

## Abbreviations

Pn: Photosynthetic rate
Gs: Stomatal conductance
Ci: Intercellular CO_2_ concentration
Tr: Transpiration rate
Fv/Fm: Maximum quantum yield of photosystem II
NPQ: Non-photochemical quenching
DEGs: Differentially expressed genes
DAMs: Differentially accumulated metabolites
ABA: Abscisic acid
