# Supplementary figures and images for: Grapevine (Vitis vinifera) responses to salt stress and alkali stress: transcriptional and metabolic profiling
Source: BMC Plant Biol. 2022 Nov 14;22:528. doi: 10.1186/s12870-022-03907-z (PMC9661776; doi:10.1186/s12870-022-03907-z)

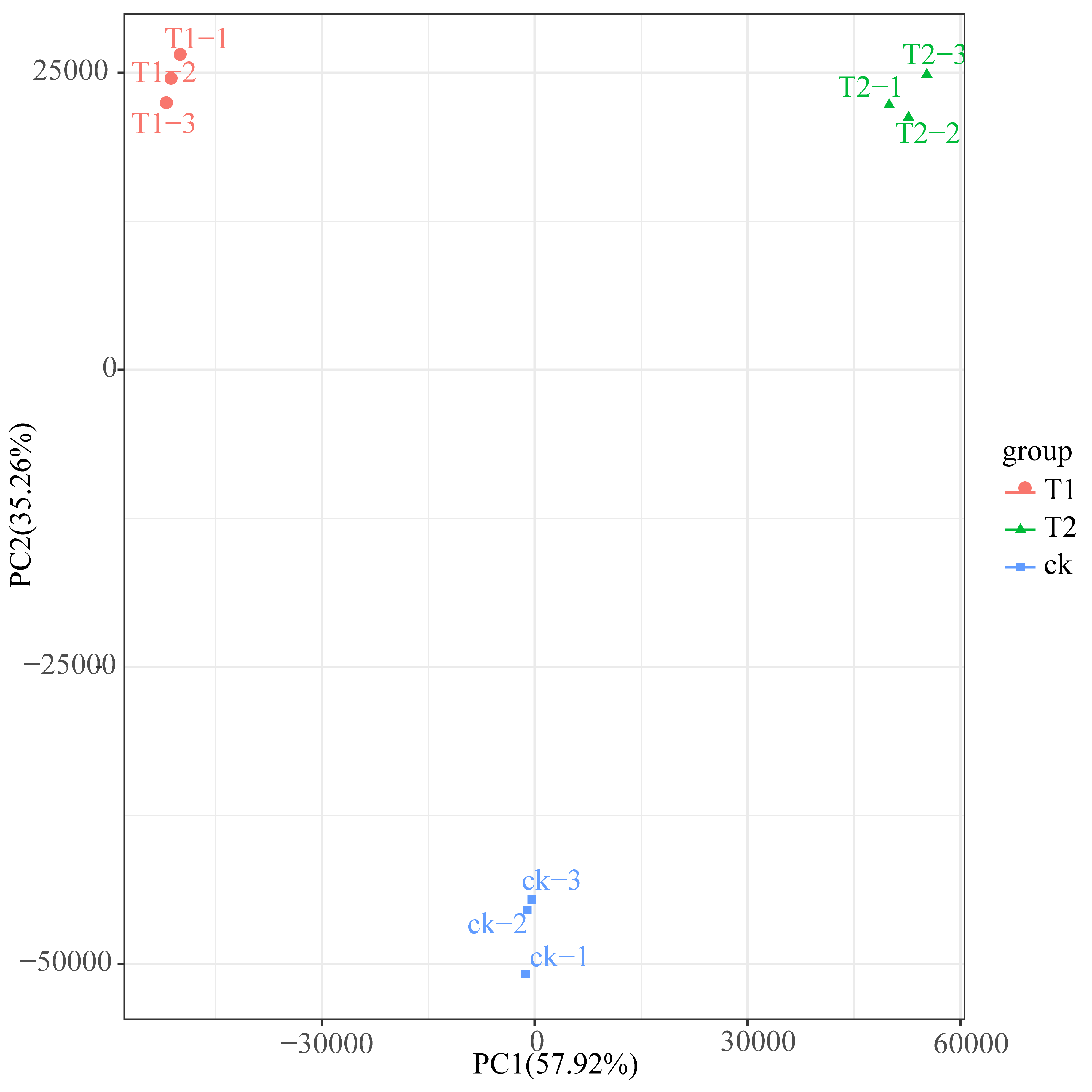

Supplement: Supplementary file 1 — Additional file 1: Figure S1. The principal component analysis (PCA) plot for DAMs. [file 12870_2022_3907_MOESM1_ESM.tif]
