# Supplementary material for: Grapevine (Vitis vinifera) responses to salt stress and alkali stress: transcriptional and metabolic profiling
Source: BMC Plant Biol. 2022 Nov 14;22:528. doi: 10.1186/s12870-022-03907-z (PMC9661776; doi:10.1186/s12870-022-03907-z)
Supplement: Supplementary file 2 — Additional file 2: Table S1. Primer sequences used in qRT-PCR analysis. [file 12870_2022_3907_MOESM2_ESM.docx]

| Table S1 Primer sequences used in qRT-PCR analysis | | |
| --- | --- | --- |
| Gene name | Primer sequenes (5’ to 3’) | Accession |
| VIT_10s0003g02900 | Forward: TCAGCGAGGGTGGACTTGACTAC  Reverse: CCTCAACGGCTCCCATCAAGATAAC | XM_002275039.3 |
| VIT_07s0005g04400 | Forward: GTGAGGCTGACCCAATCAAGAGATG  Reverse: CATCAACTGACTGGAGAACCCCTTC | XM_002271630.4 |
| VIT_00s0904g00010 | Forward:AAGCCCAAAGACGAGAAGAAATCCC  Reverse: TGCCGCATGGTCCAAATCACTG | XM_002275588.3 |
| VIT_04s0008g05590 | Forward: ATGGCTAACATGATCGTGGCTTCC  Reverse: GCGGATTTGAGGGAGAGGAGTTTG | XM_019219515.1 |
| VIT_06s0004g03550 | Forward: TGGCTGGAGTTGTTGCTGTTGAG  Reverse: ATCGCATCCTTTGGTGGCATCTG | XM_010652797.2 |
| VIT_00s0317g00050 | Forward: TCTCCTCCTCTCGCTCCTCCTC  Reverse: AAAGCCTGCTCTGAACCGTCATTAG | XM_034849508.1 |
| VIT_13s0019g03170 | Forward: CGGCAGCTTCTTGAGGAGGTTC  Reverse: GGACTTCACATCGGGCTTCTTGG | XM_019224594.1 |
| VIT_08s0040g00540 | Forward: CTAGTGTTGATCCCTGGGTGTTTCG  Reverse: GCCTTGGTCAGAGTTTCGGTGTC | XM_002283623.4 |
| VIT_01s0011g06560 | Forward: AAGATGGCTGGCTGTTGCTTGG  Reverse: GCCTGCTGAAATGTCCGAGATCC | XM_010661515.2 |
| VIT_17s0000g03690 | Forward: CACAACAGATCGCCAGGATACTACG  Reverse: TGGCTTCTTGCACCTCCTTAATCAC | XM_002276931.4 |
| VIT_06s0004g05460 | Forward: GCACGGAATATAGGAGCATCAGGAG  Reverse: CAGTTGGCGACGACGATCTTCTC | XM_002282572.3 |
| VIT_13s0019g02200 | Forward: CATTCCTCTGTCATCCGACCACAAG  Reverse: ACATGGCTAGGACTCCGAGAACC | XM_002282667.3 |
| actin-101 | Forward: ACAACGGCGGAACGGGAAATC  Reverse: CAGAACTGCTCTTGGCGGTCTC | XM_002265440.3 |
